# Increasing glycolysis by deletion of *kcs1* and *arg82* improved S-adenosyl-l-methionine production in *Saccharomyces cerevisiae*

**DOI:** 10.1186/s13568-021-01179-8

**Published:** 2021-01-19

**Authors:** Hailong Chen, Nianqing Zhu, Yan Wang, Xinxin Gao, Yuhe Song, Jia Zheng, Jiaping Peng, Xin Zhang

**Affiliations:** Jiangsu Key Laboratory of Chiral Pharmaceuticals Biosynthesis, College of Pharmacy and Chemistry & Chemical Engineering, Taizhou University, 93 Ji Chuan Road, Taizhou, 225300 People’s Republic of China

**Keywords:** Increasing glycolysis, Inositol pyrophosphates metabolism, S-adenosyl-l-methionine, *Saccharomyces cerevisiae*

## Abstract

Reprogramming glycolysis for directing glycolytic metabolites to a specific metabolic pathway is expected to be useful for increasing microbial production of certain metabolites, such as amino acids, lipids or considerable secondary metabolites. In this report, a strategy of increasing glycolysis by altering the metabolism of inositol pyrophosphates (IPs) for improving the production of S-adenosyl-l-methionine (SAM) for diverse pharmaceutical applications in yeast is presented. The genes associated with the metabolism of IPs, *arg82*, *ipk1* and *kcs1*, were deleted, respectively, in the yeast strain *Saccharomyces cerevisiae* CGMCC 2842. It was observed that the deletions of *kcs1* and *arg82* increased SAM by 83.3 % and 31.8 %, respectively, compared to that of the control. In addition to the improved transcription levels of various glycolytic genes and activities of the relative enzymes, the levels of glycolytic intermediates and ATP were also enhanced. To further confirm the feasibility, the *kcs1* was deleted in the high SAM-producing strain Y*mls1*ΔGAPmK which was deleted malate synthase gene *mls1* and co-expressed the Acetyl-CoA synthase gene *acs2* and the SAM synthase gene *metK1* from *Leishmania infantum*, to obtain the recombinant strain Y*mls1*Δ*kcs1*ΔGAPmK. The level of SAM in Y*mls1*Δ*kcs1*ΔGAPmK reached 2.89 g L^−1^ in a 250-mL flask and 8.86 g L^−1^ in a 10-L fermentation tank, increasing 30.2 % and 46.2 %, respectively, compared to those levels in Y*mls1*ΔGAPmK. The strategy of increasing glycolysis by deletion of *kcs1* and *arg82* improved SAM production in yeast.

## Key points

1. Metabolic reprogramming glycolysis through deletion of *kcs1* and *arg82* was applied for improvement of SAM production.
2. Several glycolytic genes expression levels and relative enzyme activities were enhanced by deletion of *kcs1* and *arg82*.
3. The levels of ATP and precursors were both enhanced for SAM synthesis.
4. The SAM production of Y*mls1*Δ*kcs1*ΔGAPmK reached 8.86 g L^− 1^ in a 10-L fermentation tank, increasing 46.2 %, compared to that of Y*mls1*ΔGAPmK.

## Introduction

S-adenosyl-l-methionine (SAM), an important biochemical molecule, exhibits great potential for the clinical therapy of osteoarthritis, liver disorders, depression, and Alzheimer’s disease (Fontecave et al. 2004; Cederbaum et al. 2010; Linnebank et al. 2010). Because various promising therapeutic results have increased the demand for SAM, continued efforts have been undertaken to improve the microbial production of SAM by conventional strain breeding, fermentation process optimization and the metabolic engineering of microorganisms (Choi et al. 2009; Chu et al. 2013; Kant et al. 2014).

Conventional strain breeding technologies, including screening, chemical or physical mutagenesis, composite mutagenesis, and spaceflight culture, have been used to obtain SAM productive microorganism strains (Shobayashi et al. 2006; Huang et al. 2012). The fermentation process optimization has focused on the addition of precursor amino acids, auxiliary energy substrates, the use of surfactants, and the control of pH, dissolved oxygen or ethanol production during the fermentation process (Mincheva et al. 2002; Chen et al. 2016a). One typical example of microbial production of SAM was reported by Shiozaki, who used the strain *Saccharomyces sake* Kyokai no. 6, isolated from wild strains, to produce 10.8 g L^−1^ of SAM in a 10-L bioreactor under optimized incubation conditions, which is the maximum production of SAM by a wild-type yeast strain reported to date (Shiozaki et al. 1984). Currently, strains of *Saccharomyces cerevisiae* (*S. cerevisiae*), *Pichia pastoris* (*P. pastoris*), *Candida utilis*, *Kluyveromyces lactis*, *Corynebacterium glutamicum* and *Escherichia coli*, with superior SAM productivity, have been screened and used extensively for SAM microbial production (Li et al. 2007; Wang et al. 2012; Chen et al. 2016a; Qin et al. 2020). The metabolic engineering strategies for SAM production have mainly covered the following several aspects: enhancing methionine adenosyltransferase (MAT) activity, deleting the cystathionine synthetase gene to block the transformation of SAM to cysteine, releasing the feedback inhibition of SAM to MAT and methylenetetrahydrofolate reductase (Roje et al. 2002; Chen et al. 2016a, c). In addition, it was reported that the cystathionine β-synthase gene *CYS4* of *Pichia pastoris* was downregulated using a weak promoter P_G12_ to reduce the removal of homocysteibuqianne from SAM cycle, and led to a 48.8 % increase in the SAM titer (1.68 g L^−1^) in shake flask culture. Subsequently, the SAM titer of G12-CBS was improved to 13.01 g L^−1^ in 15-L fed-batch fermentation using the optimal l-methionine feeding strategy (Qin et al. 2020). Metabolic engineering strategies that increase the availabilities of ATP and co-factor NADH for SAM production have also been reported in recent decades (Kant et al. 2014; Chen et al. 2016c). For example, a production of 9.73 g L^−1^ SAM was achieved in a 3.7-L bioreactor by the enhancements of ATP and precursor levels through co-expressing *mup1*, *adk1* and *sam2* along with building high cell density culture (Kant et al. 2014). According to the above existing reports, the microbial production of SAM usually ranges from a few grams to more than ten grams (Shiozaki et al. 1984; Chu et al. 2013; Chen et al. 2016c), and it has been shown that metabolic engineering strategy is one of important tools for directing metabolic flux towards SAM biosynthesis.

**Fig. 1:**
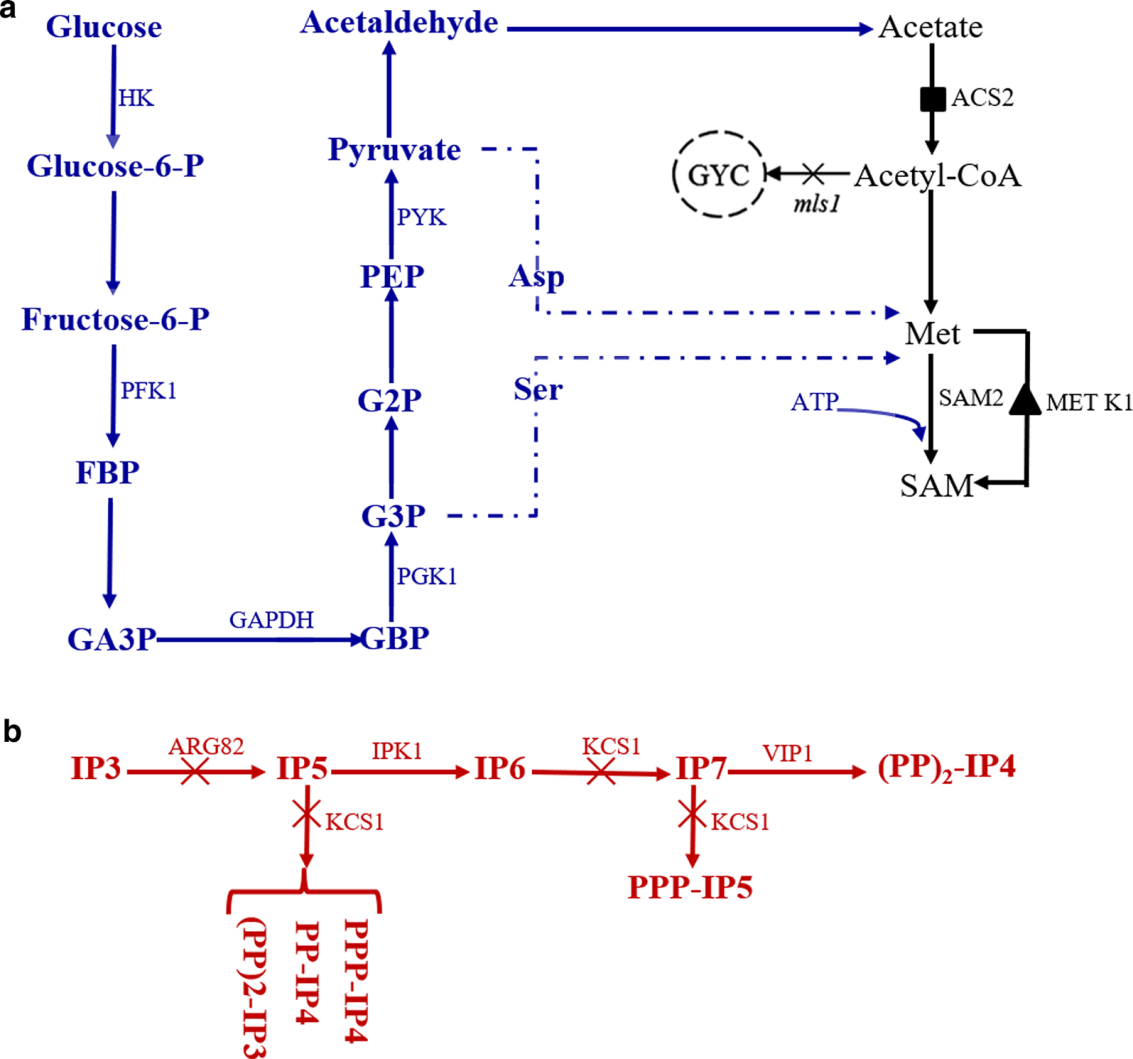
Schematic representation of increasing glycolysis by deletions of *kcs1* and *arg82* improved SAM production in *Saccharomyces cerevisiae*. **a** The glycolytic intermediates are linked to SAM metabolism; **b** Pathway for the synthesis of inositol polyphosphates. The color of blue represents the glycolysis pathway enhanced by deletions of *kcs1* and *arg82*; The color of red represents the IPs metabolism. The black triangle represents the introduction of heterologous gene; The black rectangle represents gene over-expression; The red cross represents gene deletion

Glycolysis plays a key role in carbon metabolism and usually is a target of metabolic engineering (Masumoto et al. 2018). As illustrated in Fig. 1a, glycolysis catabolizes glucose as a carbon source and synthesizes pyruvate, various glycolytic intermediates and adenosine triphosphate (ATP) in yeast (Lin et al. 2001; Nielsen et al. 2016). The glycolytic intermediates are linked to amino acid metabolism, such as l-aspartate (l-Asp), l-serine (l-Ser) and l-methionine (l-Met) (Cherest et al. 1969). Among these intermediates, pyruvate (PY) is converted into Acetyl-CoA, which is metabolized in the tricarboxylic acid cycle, or converted into l-Met or l-Asp. 3-phosphoglycerate (G3P) is converted into l-Ser through a multistep reaction. l-Asp and the l-Ser participate in l-Met metabolism through different pathways (Cherest et al. 1969; Thomas et al. 1997). Therefore, metabolic reprogramming of glycolysis may be a strategy with great potential for improving SAM synthesis in yeast.

Several glycolytic genes in *Saccharomyces cerevisiae* are tightly regulated by the transcriptional factors of GCR1, GCR2 and RAP1 (Santangelo 2006). When the intracellular levels of the inositol pyrophosphates (IPs) increase, the pyrophosphorylation of GCR1 weakens the interaction between RAP1/GCR1 and GCR2 and then represses transcription of the glycolytic genes. The absence of pyrophosphorylation of GCR1 increases transcriptional complex stability and then activates the transcription of glycolytic genes (Saiardi et al. 2004; Santangelo 2006; Szijgyarto et al. 2011). In short, the expression levels of the glycolytic genes are controlled by the GCR1 pyrophosphorylation which depends on the intracellular levels of IPs.

IPs in yeast are synthesized by the phosphorylation of inositol trisphosphate (IP_3_) (EI Alami et al. 2003). As shown in Fig. 1b, the IP_3_ is sequentially phosphorylated by serial enzymatic reactions under the catalysis of inositol phosphate multikinase (ARG82 and IPK1) to inositol tetrakisphosphate (IP_4_), inositol pentakisphosphate (IP_5_) and inositol hexakisphosphate (IP_6_). The IP_6_ is then phosphorylated by inositol pyrophosphate kinase (KCS1, encoded by *kcs1*) to IP7 or PPP-IP5, or is phosphorylated by KCS1 together with IP_7_ kinase (VIP1, encoded by *vip1*) to the double-pyrophosphate form (PP)_2_-IP_4_ of IP_8_ in yeast. The IPs species diphosphoinositol tetrakisphosphate (PP-IP_4_), PPP-IP4 and double-diphosphoinositol trisphosphate (PP)_2_-IP_3_ can be produced from IP_5_ under the catalysis of KCS1. IPs have been linked to various biological functions including vesicular trafficking, apoptosis, and cellular energetic metabolism (EI Alami et al. 2003; Bennett et al. 2006). Szijgyarto et al. (2011) deleted the *kcs1*, the *ipk1* and the *arg82*, and it was demonstrated that the glycolytic flux and the cellular ATP content can be increased by the deletion of genes related to IP-metabolism.

In our previous study, it was found that elevation of the intracellular Acetyl-CoA level by overexpressing *acs2* (encoding Acetyl-CoA synthase) and deleting *mls1* (encoding malate synthase) increased methionine synthesis and then improved SAM production in yeast. To eliminate feedback inhibition of SAM synthase and direct the metabolic flux towards SAM biosynthesis, a codon-optimized *metK1* encoding SAM synthase (MET K1) from *Leishmania infantum* was introduced in an *acs2* overexpression and *mls1* deletion strain to obtain the high SAM-producing strain Y*mls1*ΔGAPmK, in which 2.22 g L^− 1^ of SAM accumulated, which was 3.36-fold that in the yeast strain *S. cerevisiae* CGMCC 2842 (2842) (Chen et al. 2016c). Inspired by the research results of Szijgyarto et al. (2011), metabolic reprogramming glycolysis through altering the metabolism of IPs was investigated in this study for further improving the microbial production of SAM. The genes *arg82*, *ipk1*, and *kcs1* were deleted, respectively, in the yeast strain 2842. Additionally, the *kcs1* was deleted in the high SAM-producing strain Y*mls1*ΔGAPmK to obtain the new recombinant strain Y*mls1*Δ*kcs1*ΔGAPmK. Subsequently, SAM accumulation, dry cell weight (DCW) and glucose consumption were measured to verify the relationship between the metabolism of SAM and the metabolism of IPs. To further clarify the mechanism of improving SAM synthesis by *kcs1* and *arg82* deletion in yeast, the expression levels of several glycolytic genes and the relative enzyme activities were analyzed, for example, hexokinase (HK, encoded by *hxk2*), 6-phosphofructo-1-kinase (PFK1, encoded by *pfk1*), glycerol-3-phosphate dehydrogenase (GAPDH, encoded by *gapdh*), 3-phosphoglycerate kinase (PGK1, encoded by *pgk1*) and pyruvate kinase (PYK, encoded by *pyk*). In addition, the intracellular levels of ATP, precursor amino acids and various glycolytic intermediates were also investigated. This research may facilitate the improvement of the microbial production of SAM.

## Materials and methods

### Plasmids and strain construction

All plasmids, strains and oligonucleotides are summarized in Table 1. *Saccharomyces cerevisiae* CGMCC 2842 (2842) was obtained from China General Microbiology Culture Collection Center (Beijing, China) (Cao et al. 2012). The high SAM-producing strain Y*mls1*ΔGAPmK were obtained from our previous study (Chen et al. 2016c). For constructions of the *arg82* (GenBank: NC_001136.10), *ipk1* (GenBank: NC_001136.10), and *kcs1* (GenBank: NC_001136.10) deletion strains, the short flanking homology regions (SFH) replacement method and the *loxP*-*KanMx*-*loxP* method, described in a previous study, were used (EI Alami et al. 2003; Hegemann et al. 2006). The gene disruption cassettes of *loxP*-*kanMx*-*loxP* were obtained by PCR amplification using pUG6 as the template, and the G418 resistance gene marker was rescued by transforming the plasmid pSH65 with bleomycin resistance into positive transformants and inducing Cre recombinase expression by D-galactose. The primer pairs of A & B and C & D were used for deletion of *kcs1*, the primer pairs of E & F and G & H were used for deletion of *arg82*, and the primer pairs of I & J and K & L were used for deletion of *ipk1*.

**Table 1:** Strains, plasmids and primers

Strains, plasmids and primers	Relavant characteristics	Source
Strains		
2842	*S. cerevisiae* CGMCC 2842, wild type strain	Cao et al. (2012)
Y*mls1*△GAPmK	*S. cerevisiae* CGMCC 2842 derivative, *mls1*△, containing pGAL1-*acs2*- pPGK1-*metK1*	Chen et al. (2016c)
YGK	*S. cerevisiae* CGMCC 2842 derivative, containing pYES-KanMX	This study
Y*arg82*△	*S. cerevisiae* CGMCC 2842 derivative, *arg82*△	This study
Y*ipk1*△	*S. cerevisiae* CGMCC 2842 derivative, *ipk1*△	This study
Y*kcs1*△	*S. cerevisiae* CGMCC 2842 derivative, *kcs1*△	This study
Y*kcs1*△*arg82*△	*S. cerevisiae* CGMCC 2842 derivative, *kcs1*△, *arg82*△	This study
Y*kcs1*△pG-*kcs1*	Y*kcs1*△ derivative, containing pGAL1-*kcs1*	This study
Y*arg82*△pG-*arg82*	Y*arg82*△ derivative, containing pGAL1-*arg82*	This study
Y*mls1*△*kcs1*△GAPmK	*S. cerevisiae* CGMCC 2842 derivative, *mls1*△, *kcs1*△, containing pGAL1-*acs2*- pPGK1-*metK1*	This study
Plasmids		
pYES 2.0	2 µ, URA3	Invitrogen
pYES-KanMX	pYES 2.0 derivative, 2 µ, G418 resistance gene	Cao et al. (2012)
pGAL1-*kcs1*	pYES-KanMX derivative, 2 µ, G418 resistance, expression of *kcs1* of *Saccharomyces cerevisiae*	This study
pGAL1-*arg82*	pYES-KanMX derivative, 2 µ, G418 resistance, expression of *arg82* of *Saccharomyces cerevisiae*	This study
pGAL1-*acs2*-pPGK1-*metK1*	pYES-KanMX derivative, 2 µ, G418 resistance, expression of *acs2* of *S. cerevisiae* and *metK1* of *Leishmania infantum* under control of GAL1 and PGK1 promoter, respectively	Chen et al. (2016c)
pUG6	Template plasmid containing loxP-KanMX-loxP elements	Euroscarf
pSH65	Cre containing plasmid for loxP-KanMX-loxP cassette recycle	Euroscarf
Primers		
* kcs1*F	cggggtacc ATGGATACCTCTCACGAAATTCATGA	This study
* kcs1*R	cgagctc TCAATCACTAACTTGAGCATCGTC	This study
* arg82*F	cggggtacc ATGGATACGGTAAACAATTATAGG	This study
* arg82*R	cgagctc CTAGAATTTCATAAAAATATCTAGC	This study
A	attctgttcttgtttgtctctgttggtt ggattatatctcc TACGCTGCAGGTCGACAAC	This study
B	cctcacacgtccgagctcttcatcagtc ctattcctacgc TAGGCCACTAGTGGATCTG	This study
C	tggatacctctcacgaaattcatgata aaatacccgatac TACGCTGCAGGTCGACAAC	This study
D	caatcactaacttgagcatcgtcattgtatcttggttcag TAGGCCACTAGTGGATCTG	This study
E	atggatacggtaaacaattatagggttttagagcataaag TACGCTGCAGGTCGACAAC	This study
F	ctagaatttcataaaaatatctagcaaggtttcaactcct TAGGCCACTAGTGGATCTG	This study
G	tggccacgacggtactctaacagacggtgatggattgctc TACGCTGCAGGTCGACAAC	This study
H	tcacgttttcatcataacccttccccggcgttattttcaga TAGGCCACTAGTGGATCTG	This study
I	atgcaagtcatcggacgtggtggggcaaatatactgattg TACGCTGCAGGTCGACAAC	This study
J	atggggatcctacgtggttgtggcgatgctgcatccgttg TAGGCCACTAGTGGATCTG	This study
K	ttatttatttgaagtatgataaattttttggcttgatgtc TACGCTGCAGGTCGACAAC	This study
L	ggctgtaagtttttgtccaatgggtccatttttcttttgg TAGGCCACTAGTGGATCTG	This study

A 3.1 kb *Kpn* I/*Sac* I fragment including *kcs1* and a 1 kb *Kpn* I/*Sac* I fragment including *arg82* were PCR-amplified from the chromosomal DNA of the yeast strain 2842 and then inserted into corresponding site of pYES-KanMx to obtain pGAL1-*kcs1* and pGAL1-*arg82*.

All plasmids and gene disruption cassettes were transformed into 2842 using the lithium acetate method with G418 resistance selection (Amberg et al. 2005). All of the engineered strains were verified by DNA sequencing.

### Media and culture conditions

All yeast strains were streaked onto YPD solid medium from glycerol stocks and incubated at 30 °C for 20 h. The colonies were transferred into 50 mL of YPD medium and incubated for 20 h at 30 °C and 200 rpm. To ensure the plasmid was not lost in the recombinant strains, 250 µg mL^−1^ G418 was added into the YPD plates and medium to maintain a selection pressure. The YPD medium was comprised of 1.0 % peptone, 2.0 % yeast extract and 2.0 % glucose. The YPD solid medium contained YPD medium plus 2.0 % agar. A total of 2.5 mL of the seed culture (10^7^ CFU mL^−1^) was added to 50 mL of the O-medium and cultured for 48 h at 30 °C and 200 rpm. D-galactose (2 %) was added to the medium at 16 h to induce expression of the target genes. The O-medium contained 5.0 % glucose, 0.5 % yeast extract, 1.0 % peptone, 0.05 % MgSO_4_·7H_2_O, 0.4 % KH_2_PO_4_, 0.2 % K_2_HPO_4_, and 0.15 % l-Met, pH 6.0 (Chen et al. 2016b, c). Samples were taken every 4 h to determine the relevant parameters.

The SAM accumulation capacity of recombinant yeast strain Y*mls1*Δ*kcs1*ΔGAPmK was assessed in a 10-L fermenter containing 7 L of O-medium (B. Braun Biotech, Melsungen, Germany). A 500 mL inoculum was transferred into the fermenter. The culture conditions were 30 °C and pH 6.0, and the dissolved oxygen was real-timely monitored, and was maintained at a level of about 30 % by automatic control of the ventilation rate and stirring rate. Samples were taken every 4 h to determine the biomass, SAM production and reducing sugar concentration. After 12 h of fermentation, the concentration of reducing sugar was low to 5 g/L. Then, the molasses was fed at a rate of 1.2 g L^− 1^ h^− 1^ for 72 h. Then, 2 % D-galactose was added to the fermentation broth at 84 h to induce gene expression for SAM accumulation. The whole fermentation process continued for 140 h (Chen et al. 2016c, 2020).

### Dry cell weight and glucose concentration determination

The DCW and glucose concentration were analyzed every 4 h. The DCW was analyzed according to the methods reported by Chen et al. (2016b). The glucose concentration was determined by the 3,5-dinitrosalicylic acid method (Miller 1959).

### SAM, l-Met, l-Asp and l-Ser level analyses

SAM, l-Met, l-Asp and l-Ser levels were quantified by using a Shimadzu LC10A HPLC system (Shimadzu, Kyoto, Japan) equipped with a Megres C18 column (5 µm, 4.6 mm × 250 mm) (Hanbon Sci. & Tech., China). Peak area analysis was performed based on the standard calibration curves of SAM, l-Met, l-Asp and l-Ser (Sangon, Shanghai, China).

For SAM level analysis, 1.0 mL fermentation broth was mixed with 2.0 mL of 1.5 mol L^−1^ perchloric acid and stored at 40 °C for 0.5 h. The supernatant was filtered through 0.22 µm filtering discs after centrifugation at 8 000 rpm for 10 min. Then, 10 µL of sample was injected into the HPLC system. The mobile phase was 0.15 mol L^− 1^ ammonium formate (pH 3.0) with a flow rate of 1.0 mL min^−1^, and detection was monitored at 254 nm (Chen et al. 2016b, c).

For l-Met level analysis, 1.0 mL fermentation broth at the end of the logarithmic phase was sampled and centrifuged at 8000 rpm for 10 min. The cells were mixed with 2.0 mL 1.5 mol L^−1^ perchloric acid and stored at 40 °C for 0.5 h. The supernatant was filtered through 0.22 µm filtering discs after centrifugation at 8000 rpm for 10 min. The mobile phase was 10 % (v/v) methanol, and detection was monitored at 210 nm. All other conditions were the same as for the SAM analysis (Chen et al. 2016b, c).

For the l-Asp level analysis, the mobile phase was a mixture of 95 % phosphate buffer solution (0.03 mol L^−1^, pH 2.5) and 5 % methanol. The flow rate of the mobile phase was 1.0 mL min^− 1^, the sample volume was 10 µL, and the detection wavelength was 210 nm. All other conditions were the same as for the l-Met analysis (Zhang et al. 2007).

The l-Ser level was assayed by the HPLC method reported by Hagishita et al. (1996). Elution was performed with a mixture of 0.5 mmol L^− 1^ CuSO_4_, 10 mmol L^−1^ 1-heptanesulfonic acid and 1.0 mmol L^−1^ KH_2_PO_4_. The flow rate of the buffer was 1.0 mL min^−1^, and l-Ser was detected at 230 nm as chelated complexes with copper ions.

### Intracellular glycolytic intermediates level analyses

For the analyses of glucose-6-phosphate (G6P), fructose-1,6-bisphosphate (FBP) and pyruvate (PY), the samples were taken at the end of the logarithmic phase and prepared as follows: 2.0 mL fermentation broth was centrifuged at 8000 rpm for 10 min. The cells were mixed with 2.0 mL of 1.5 mol L^−1^ perchloric acid and stored at 40 °C for 30 min. After that, the mixture was centrifuged at 8000 rpm for 5 min to remove proteins and then the supernatant was collected. Next, the supernatant was neutralized with 2.5 mol L^− 1^ K_2_CO_3_ at 40 °C (Zhu et al. 2011; Chen et al. 2016b).

The intracellular level of G6P was assayed by the procedure reported by Zhu et al. (2011). The assay procedure was as follows: 10 µL of the G6P standards (0, 0.2, 0.4, 0.6, 0.8, 1.0 µmol L^−1^) and extraction samples were added to a 96-well plate, followed by the addition of 90 µL of an assay mixture containing 50 mmol L^−1^ triethanolamine (pH 7.6), 1.0 mmol L^−1^ MgCl_2_, 100 µmol L^− 1^ NADP^+^, 10 µmol L^− 1^ resazurin, 0.1 U mL^− 1^ G6P dehydrogenase (One unit (U) reduces 1.0 µmol of NAD^+^ per minute at 37 °C, pH 7.8, using glucose-6-phosphate as substrate) (Sangon, Shanghai, China), and 0.2 U mL^−1^ diaphorase (One unit equals a decrease in absorbance at 600 nm of 1.0 per minute at 25 °C, pH 7.5) (Sangon, Shanghai, China). These mixtures were incubated at room temperature for 30 min. Fluorescence at 590 nm was measured using excitation at 530 nm. Background fluorescence was corrected by subtracting the value of the no-G6P control from all sample readings. Fluorescence was measured using a FlexStation II plate reader (Molecular Devices, Sunnyvale, CA, USA).

The assay procedure of FBP was as follows: 1 mL of the extract sample solution was mixed with 2 mg resorcinol and 7 mL hydrochloric acid, incubated at 80 °C for 15 min, and then cooled, followed by measurement of the absorbance at 570 nm (Du et al. 1993).

The intracellular level of PY was assayed enzymatically with lactic acid dehydrogenase (Li et al. 2001; Saavedra et al. 2008). The reaction mixture for the assay consisted of 300 µmol potassium phosphate buffer (pH 7.0), 0.05 U lactic acid dehydrogenase (One unit lactic acid dehydrogenase oxidizes 1.0 µmol of NADH per minute at 25 °C, pH 7.3) (Sangon, Shanghai, China), 0.3 µmol NADH, and 100 µL of diluted extract sample solution in a final volume of 3.0 mL. The decrease in NADH concentration, measured by the change in absorbance at 340 nm, was proportional to the amount of PY reduced.

### Intracellular Acetyl-CoA and ATP levels analyses

For the analyses of intracellular levels of Acetyl-CoA and ATP, 1.0 mL fermentation broth was sampled at the end of the logarithmic phase and centrifuged at 8000 rpm for 10 min. The cells were mixed with 2.0 mL 1.5 mol · L^−1^ perchloric acid and stored at 40 °C for 0.5 h. The supernatant was filtered through 0.22 µm filtering discs after centrifugation at 8000 rpm for 10 min. The intracellular levels of Acetyl-CoA and ATP were analyzed using a Shimadzu LC10A HPLC system (Shimadzu, Kyoto, Japan) equipped with a Megres C18 column (5 µm, 4.6 mm × 250 mm) (Hanbon Sci. & Tech., China). For the Acetyl-CoA analysis,the mobile phase was 80 % buffer A (0.2 mol/L sodium phosphate pH 5.0) and 20 % buffer B (800 mL of 0.25 mol/L sodium phosphate pH 5.0 mixed with 200 mL of acetonitrile). For the ATP analysis, the mobile phase was 95 % (v/v) 0.05 mol/L sodium phosphate buffer (pH 6.0) and 5 % (v/v) methanol. All the other conditions were the same as SAM determination. The assay procedures were from previously published reports (Chen et al. 2016b, c).

### RNA extraction, reverse transcription and real‐time qPCR

For determination of the expression levels of several glycolytic genes, total RNA was isolated from yeast cells at the end of the logarithmic phase using the Total RNA Isolation Kit (Sangon, ShangHai, China). The RNA quality was verified on a 1 % agarose gel, and the concentration was measured with an Eppendorf BioPhotometer Plus (Hamburg, Germany). The same concentration of total RNA (1 µg) was reverse transcribed using the PrimeScript RT Reagent Kit with gDNA Eraser (Perfect Real Time, Takara) following the manufacturer’s instructions. The primers used for quantitative PCR and the GenBank numbers of the reference sequences were summarized in Table 2. The transcriptions of the genes were quantified by real-time qPCR on a StepOnePlus instrument (ABI, USA) by using the 2^−ΔΔCT^ algorithm (Yuan et al. 2006; Szijgyarto et al. 2011).

**Table 2:** The primers used in QPCR analyses of the expression of glycolytic genes in this study

Gene	Sense primer	Antisense primer	Refseq GenBank number
*hxk2*	ctgctccaatggccatcaac	aaggtttgttggcctggtct	NC_001139.9
*pfk1*	tggtcttgtcggttccatcg	aaggtttgttggcctggtct	NC_001139.9
*gapdh*	agtcttttgggtggcggtca	acattgacgctggtgccaag	NM_001181666.1
*pgk1*	aggcttctgccccaggttc	cagcacgttgtggcaagtc	NC_001135.5
*pyk*	cgactcagatgctggattca	ccgtttctccagaaagcataa	NC_001133.9

### Enzymatic assays

The yeast cells were sampled at the end of the logarithmic phase and harvested by centrifugation at 8000 rpm for 5 min and washed twice with a phosphate-buffered (pH 7.0) solution. Cell-free extracts were prepared by ultrasonication. The ultrasonic conditions were as followed: the total time was 10 min (on-time 10 s and off-time 10 s), the ultrasound power was 300 W at 22 kHz frequency.

The activity of HK was determined using the method reported by Panneman et al. (1998). The cell-free extract was added to the enzymatic assay mixture containing 50 mmol L^−1^ PIPES buffer (pH 7.5) in the presence of 5 mmol L^−1^ MgCl_2_, 5 mmol  L^−1^ fructose, 2 mmol L^−1^ ATP, 0.5 mmol · L^−1^ NADP^+^, 2 U mL^−1^ glucose-6-P dehydrogenase (One unit reduces 1.0 µmol of NAD^+^ per minute at 37 °C, pH 7.8, using glucose-6-phosphate as substrate) (Sangon, Shanghai, China) and 4 U mL^−1^ phosphoglucose isomerase (One unit will convert 1.0 µmol of D-fructose 6-phosphate to D-glucose 6-phosphate per minute at pH 7.4, at 25 °C) (Sigma, Saint Louis, USA) at 30 °C. The amount of consumed NADH was determined by measuring the decrease in absorbance at 340 nm. One unit of activity was defined as the amount of enzyme that formed 1 µmol NADPH per min (Panneman et al. 1998).

The activity of PFK1 was measured by the coupled enzyme assay reported by Lötscher et al. (1984). The cell-free extract was added to the enzymatic assay mixture containing 25 mmol L^− 1^ Tris (pH 6.9), 2 mmol L^−1^ ATP, 5 mmol L^− 1^ MgCl_2_, 2 mmol L^− 1^ phosphoenol pyruvate, 30 U mL^−1^ pyruvate kinase (One unit will convert 1.0 µmol of phosphoenolpyruvate acid to pyruvate acid per minute at pH 7.6, at 25 °C) (Sigma, Saint Louis, USA), 30 U mL^−1^ l-lactic dehydrogenase (One Unit oxidizes 1.0 µmol of NADH per minute at 25 °C, pH 7.3) (Sangon, Shanghai, China) and 0.5 mmol L^−1^ NADH. The reaction was started by addition of 5 mmol L^−1^ fructose-6-phospate. NADH oxidation was monitored at 340 nm for 5 min. One unit of PFK1 activity was defined as 1 mmol FBP formed per min.

The activity of GAPDH was determined using the method reported by He et al. (2014). Substrate solution (100 µL) containing triethanolamine buffer (pH 7.9), 0.5 % bovine serum albumin, 0.2 mmol L^− 1^ NADH and 1 mmol L^−1^ dihydroxyacetone phosphate was added to a 96-well plate and incubated at 37 °C for 5 min, after which 100 µL cell-free extract was added and the plate was agitated for 5 min. The amount of consumed NADH was determined by measuring absorbance at 340 nm. One unit (U) of activity was defined as the amount of enzyme that oxidized 1 µmol NADH per min at 37 °C.

For the determination of activity of PGK, the reaction solution contained 50 mmol L^−1^ potassium phosphate (pH 6.7), 5 mmol L^− 1^ MgCl_2_, 1 mmol L^−1^ EDTA, 1 mmol L^−1^ dithiothreitol, 0.7 mmol L^− 1^ NAD^+^, 1 mmol L^−1^ ADP, 1.6–3.2 U GAPDH, 0.3–30 mg protein cytosolic extract. The reaction was started with 3 mmol L^−1^ G3P. The amount of reduced NAD^+^ was determined by measuring absorbance at 340 nm (Saavedra et al. 2008).

The activity of PYK was determined in 50 mmol L^−1^ imidazole buffer (pH 7.0) containing 10 mmol L^− 1^ MgCl_2_ and 100 mmol L^−1^ KCl. The substrates ADP and phosphoenopyruvate (PEP) were used at a concentration of 2 mmol L^−1^, and the FBP was added as an activator at 1 mmol L^− 1^. The reaction was coupled to NADH oxidation by addition of 1 U mL^−1^ of lactate dehydrogenase (One Unit oxidizes 1.0 µmol of NADH per minute at 25 °C, pH 7.3) (Sangon, Shanghai, China) and 0.2 mmol L^− 1^ NADH. The reaction activity was monitored at 30 °C by measuring the decrease in absorbance at 340 nm. One unit of activity was defined as the amount of enzyme that oxidized 1 µmol NADH per min at 30 °C (Boles et al. 1997; Saavedra et al. 2008).

### Statistical analysis

All above experiments were performed three times, each time in triplicates. Statistical analysis was performed with GraphPad Prism v5.01 software, San Diego California, USA. Error bars correspond to standard error of mean (SEM) of the biological replicates (n = 3). *** denotes significant differences between the recombinant strain and the wild strain, with p < 0.001.

## Results

### Deletion of *kcs1* and *arg82* increased the glucose uptake rate and improved SAM production in *S. cerevisiae*

**Fig. 2:**
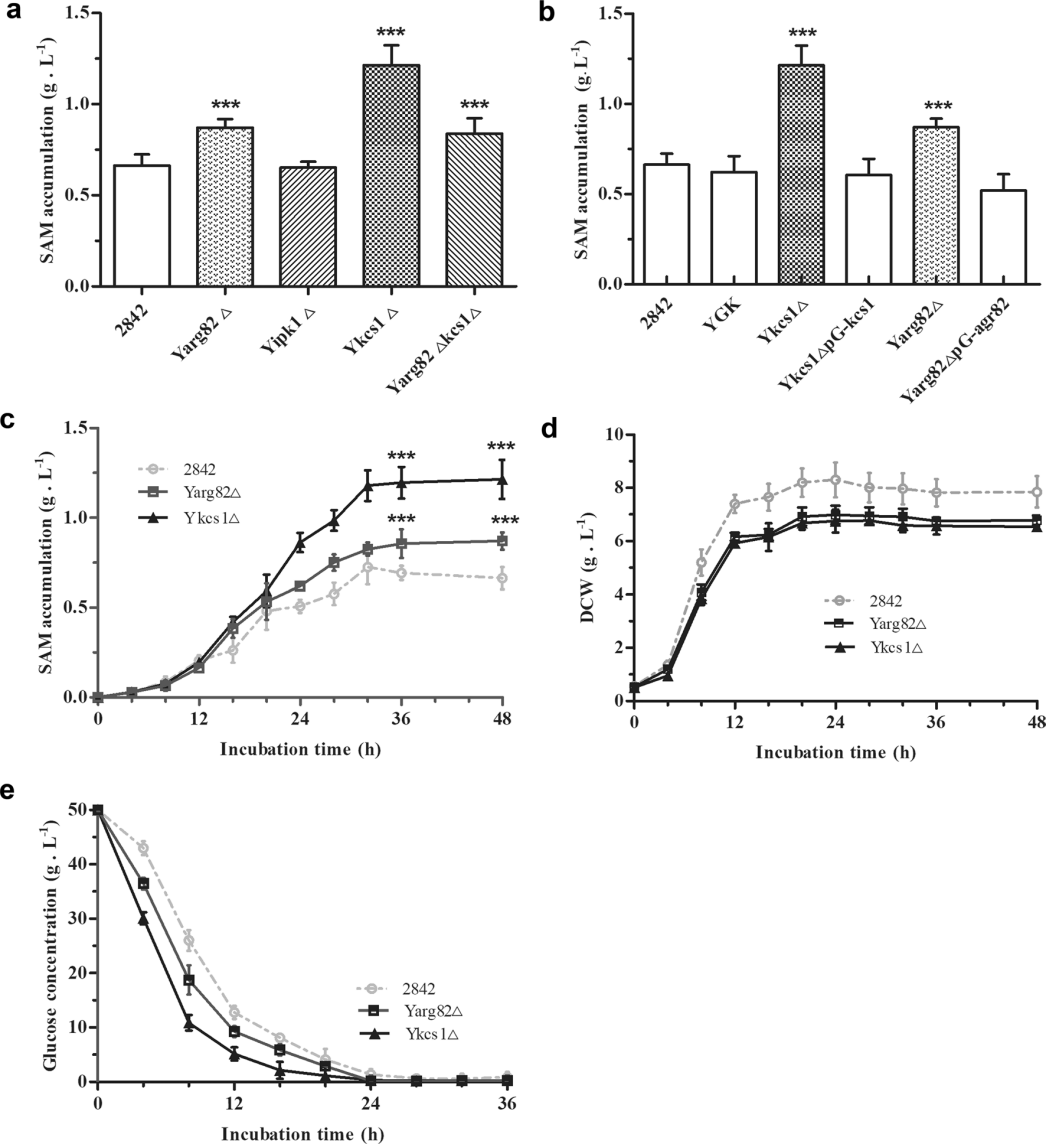
Deletion of *kcs1* and *arg82* improved SAM production and increased the glucose uptake rate in *Saccharomyces cerevisiae*. **a** The SAM accumulations of the strains deleted the several genes in metabolism of IPs; **b** The SAM accumulations of the strains reintroduced of *kcs1* and *arg82*; **c** The accumulation curve of SAM of the *kcs1* or *arg82* deletion strains; **d** The glucose consumption curves of *kcs1* or *arg82* deletion strains; **e** The growth curve of the *kcs1* or *arg82* deletion strains. Error bars correspond to standard error of mean (SEM) of the biological replicates (n = 3). *** denotes significant differences between the recombinant strain and the wild strain, with p < 0.001

To assess whether SAM biosynthesis is affected by metabolism of the IPs, the relative genes *arg82*, *kcs1* and *ipk1* were deleted in yeast strain 2842, and the SAM accumulation in the fermentation broth of the different mutant yeast strains was measured. Y*arg82*Δ, Y*kcs1*Δ and Y*arg82*Δ*kcs1*Δ accumulated 0.87 g L^−1^, 1.21 g L^−1^ and 0.84 g L^− 1^ SAM, which was 31.8 %, 83.3 % and 27.3 % higher than that in the wild type strain 2842 (0.66 g L^−1^ SAM), respectively. In contrast, the Y*ipk1*Δ strain accumulated 0.65 g L^−1^ SAM, which was not significantly different from that of 2842 (Fig. 2a and c). To further confirm this, the *arg82* gene and the *kcs1* gene were reintroduced into the mutant Y*arg82*Δ and the Y*kcs1*Δ strains, respectively. It was found that the SAM accumulation in these two strains decreased (Fig. 2b). The above research confirmed a correlation between SAM biosynthesis and the metabolism of IPs.

The DCW and glucose uptake rate were also investigated during the fermentation process. Y*arg82*Δ, Y*kcs1*Δ and 2842 accumulated 6.78 g L^−1^, 6.54 g L^−1^ and 7.85 g L^−1^ of DCW at 48 h of fermentation (Fig. 2d). It was found that the biomasses of the mutant strains were lower than that of the wild type yeast strain 2842. Conversely, the glucose uptake rates were greater than that of 2842 (Fig. 2e). In addition, both the ratio of SAM yield to DCW and the ratio of SAM yield to carbon source of Y*arg82*Δ, Y*kcs1*Δ and Y*arg82*Δ*kcs1*Δ were significantly improved compared to those of 2842, except for those of Y*ipk1*Δ (Table 3). The results demonstrated that the deletion of *kcs1* and *arg82* enhanced the microbial productivity of SAM in the mutant yeast strain. In short, altering the metabolism of the IPs through the deletion of *kcs1* and *arg82* improved SAM production, and increased the glucose uptake rate in yeast.

**Table 3:** The deletion of *kcs1* and *arg82* increased the glucose uptake rate and improved the SAM production in yeast

	2842	Y*arg82*△	Y*ipk1*△	Y*kcs1*△	Y*arg82*△*kcs1*△
SAM (g L^− 1^)	0.66 ± 0.06	0.87 ± 0.05***	0.65 ± 0.03	1.21 ± 0.11***	0.84 ± 0.09***
DCW (g L^− 1^)	7.85 ± 0.59	6.78 ± 0.19***	6.63 ± 0.22***	6.54 ± 0.16***	6.23 ± 0.12***
SAM yield to DCW (mg g^− 1^)	84.1	128.3***	98.0***	185.0***	134.8***
SAM yield to carbon source (mg g^− 1^)	13.2	17.4***	13.0	24.2***	16.8***

The symbol *** denotes significant diferences between the recombinant strain and the wild strain, with p < 0.001

### Deletion of *kcs1* and *arg82* increased the expression levels of several glycolytic genes and enzyme activities in *S. cerevisiae*

**Fig. 3:**
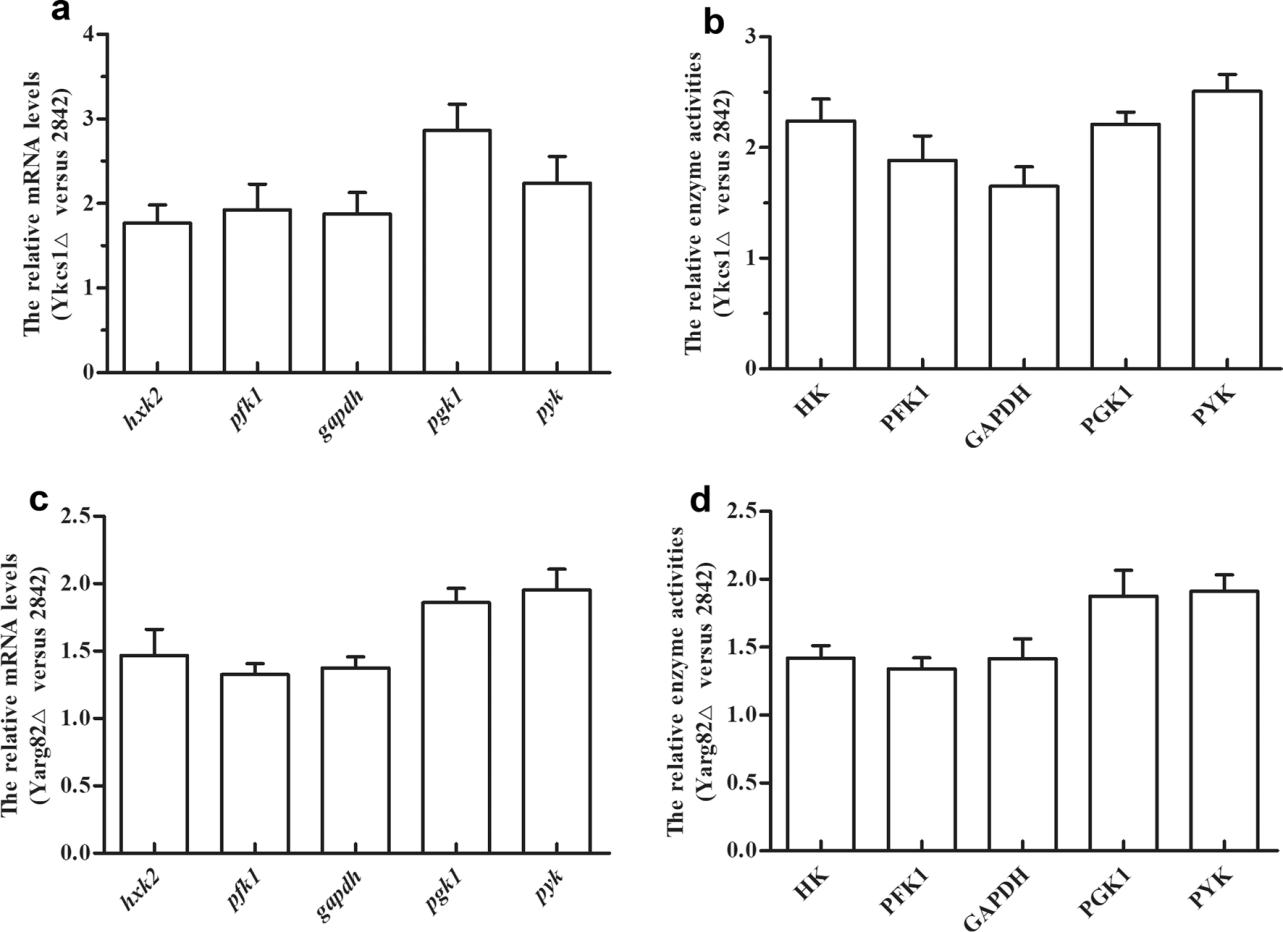
Deletion of *kcs1* and *arg82* increased the expression levels of several glycolytic genes and enzyme activities in yeast. **a** The relative expression levels of *hxk2*, *pfk1*, *gapdh*, *pgk1* and *pyk* of the *kcs1* deletion strain; **b** The relative activities of enzymes HK, PFK1, GAPDH, PGK1 and PYK of the *kcs1* deletion strain; **c** The relative expression levels of *hxk2*, *pfk1*, *gapdh*, *pgk1* and *pyk* of the *arg82* deletion strain; **d** The relative activities of enzymes HK, PFK1, GAPDH, PGK1 and PYK of the *arg82* deletion strain. Error bars correspond to standard error of mean (SEM) of the biological replicates (n = 3)

To explore the reasons for the increase of the glucose uptake, the responses of glycolysis to deletions of the *kcs1* and the *arg82* genes in the *S. cerevisiae* strain were investigated by quantitative analyses of the expression levels of several glycolytic genes and enzyme activities, as shown in Fig. 3. Compared to the wild type yeast strain 2842, the expression levels of *hxk2*, *pfk1*, *gapdh*, *pgk1* and *pyk* in the *kcs1* deletion strain Y*kcs1*Δ increased by 77 %, 93 %, 88 %, 186 % and 124 %, and the activities of the related enzymes HK, PFK1, GAPDH, PGK1 and PYK increased by 124 %, 88 %, 65 %, 121 % and 151 %, respectively (Fig. 3a and b). The same phenomenon was also observed in the *arg82* deletion strain Y*arg82*Δ (Fig. 3c and d). The results showed that the deletion of *kcs1* and *arg82* increased the expression levels of several glycolytic genes and enhanced the relative enzyme activities in yeast.

### The increase in glycolysis elevated the intracellular levels of ATP for SAM biosynthesis

**Fig. 4:**
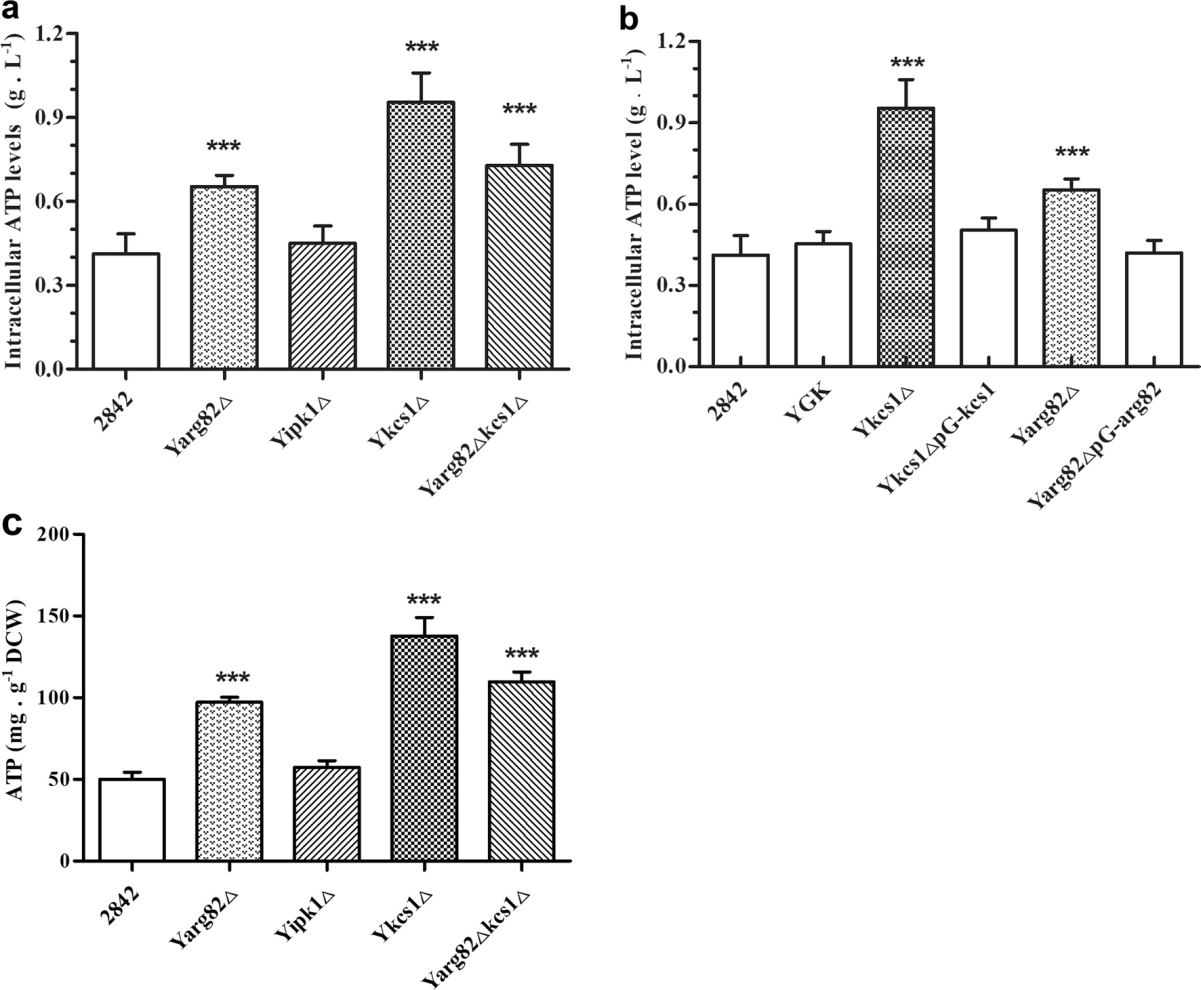
Deletion of *kcs1* and *arg82* increased the glucose uptake rate and improved SAM production in *Saccharomyces cerevisiae*. **a** The intracellular levels of ATP of the strains deleted several genes in metabolism of IPs; **b** The effects of reintroductions of *kcs1* or *arg82* on the intracellular levels of ATP. Error bars correspond to standard error of mean (SEM) of the biological replicates (n = 3). *** denotes significant differences between the recombinant strain and the wild strain, with p < 0.001

An increase in glucose consumption most likely will influence the cellular energy dynamics, and thus the intracellular ATP levels of the mutant strains were determined. It was found that Y*arg82*Δ, Y*kcs1*Δ and Y*arg82*Δ*kcs1*Δ accumulated 0.65 g L^−1^, 0.95 g L^−1^ and 0.72 g L^−1^ of ATP, which represented an increase of 59 %, 132 % and 76 %, respectively, compared to the wild type yeast strain 2842. The Y*ipk1*Δ strain accumulated 0.45 g L^−1^ of ATP, which was not significantly different from that of 2842 (Fig. 4a). Similar to the trend in SAM accumulation, the reintroduction of the *arg82* and *kcs1* genes into the mutant strains of Y*arg82*Δ and Y*kcs1*Δ decreased the intracellular levels of ATP (Fig. 4b). In addition, The ATP levels were both increased when normalized by the volume of fermentation broth and normalized by cell weight (Fig. 4a and c). Analysis of the intracellular levels of ATP in mutants deleted for various inositol phosphate kinases confirmed a correlation between the metabolism of the IPs and the increase in the intracellular levels of ATP.

### The increase in glycolysis elevated the intracellular levels of the glycolytic intermediates and l-Met precursors for SAM biosynthesis in yeast

**Fig. 5:**
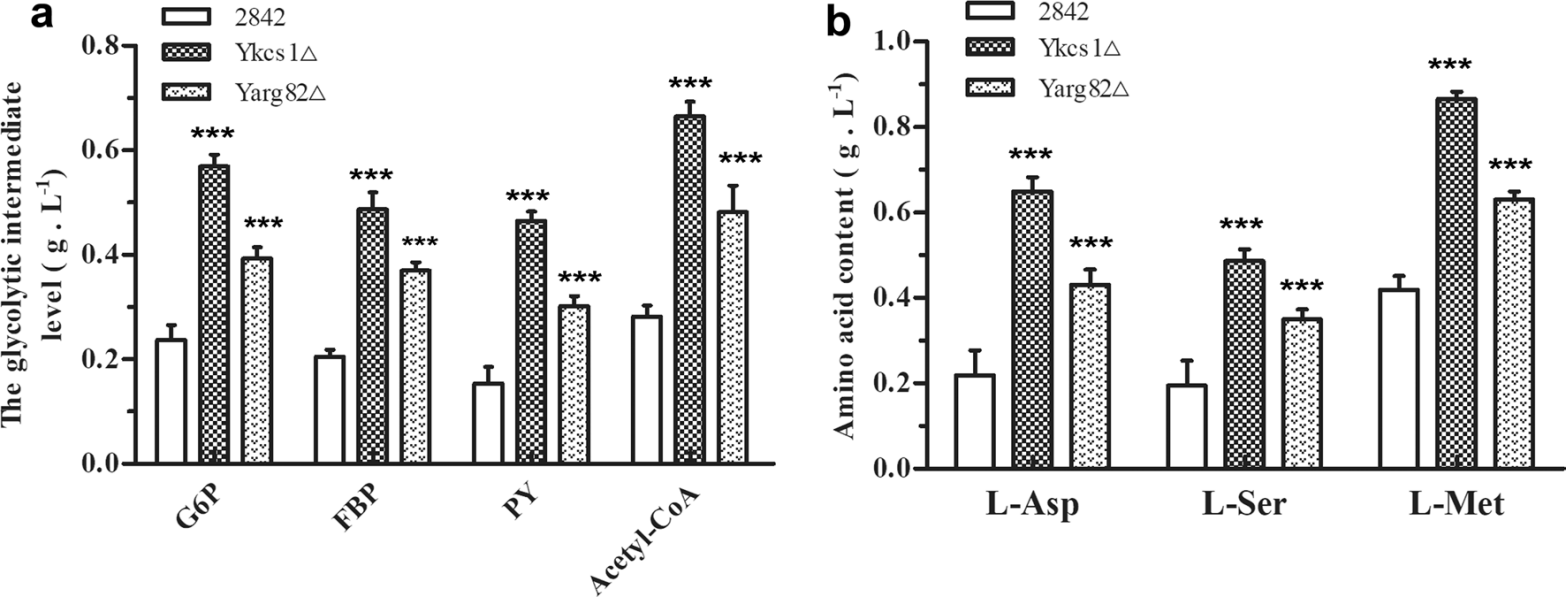
The deletion of *kcs1* and *arg82* elevated the intracellular levels of glycolytic intermediates and precursors amino acid for the biosynthesis of SAM in yeast. **a** The intracellular levels of glycolytic intermediates; **b** The intracellular levels of the precursors amino acid. Error bars correspond to standard error of mean (SEM) of the biological replicates (n = 3). *** denotes significant differences between the recombinant strain and the wild strain, with p < 0.001

In addition to energy, the precursor supply is also important for the synthesis of SAM. Quantitative analyses of the intracellular levels of several glycolytic intermediates and precursor amino acids participating in l-Met metabolism were carried out, as shown in Fig. 5. It was found that the intracellular levels of G6P, FBP and PY of the Y*kcs1*Δ strain reached 0.57 g L^−1^, 0.49 g L^−1^ and 0.47 g L^−1^, which represented increases of 138 %, 133 % and 213 %, respectively, compared to 2842 strain which accumulated 0.24 g L^−1^ of G6P, 0.21 g L^−1^ of FBP, and 0.15 g L^−1^ of PY. The intracellular levels of Acetyl-CoA, l-Asp, l-Ser and l-Met of the Y*kcs1*Δ strain reached 0.67 g L^−1^, 0.65 g L^−1^, 0.49 g L^−1^ and 0.87 g L^−1^, which represented an increase of 139 %, 195 %, 145 % and 107 %, respectively, compared to 2842 compared to 2842 strain which accumulated 0.28 g L^−1^ of Acetyl-CoA, 0.22 g L^−1^ of l-Asp, 0.20 g L^−1^ of l-Ser, and 0.42 g L^−1^ of l-Met. Similar to the Y*kcs1*Δ strain, the intracellular levels of several glycolytic intermediates and precursor amino acids participating in the metabolism of l-Met were also enhanced in the Y*arg82*Δ strain. The above results demonstrated that the deletion of *kcs1* and *arg82* increased glycolysis and elevated the intracellular levels of glycolytic intermediates and precursor amino acids for the biosynthesis of SAM in yeast.

### Increased glycolysis by deletion of *kcs1* in the recombinant strain Y*mls1*ΔGAPmK further improved SAM production

**Fig. 6:**
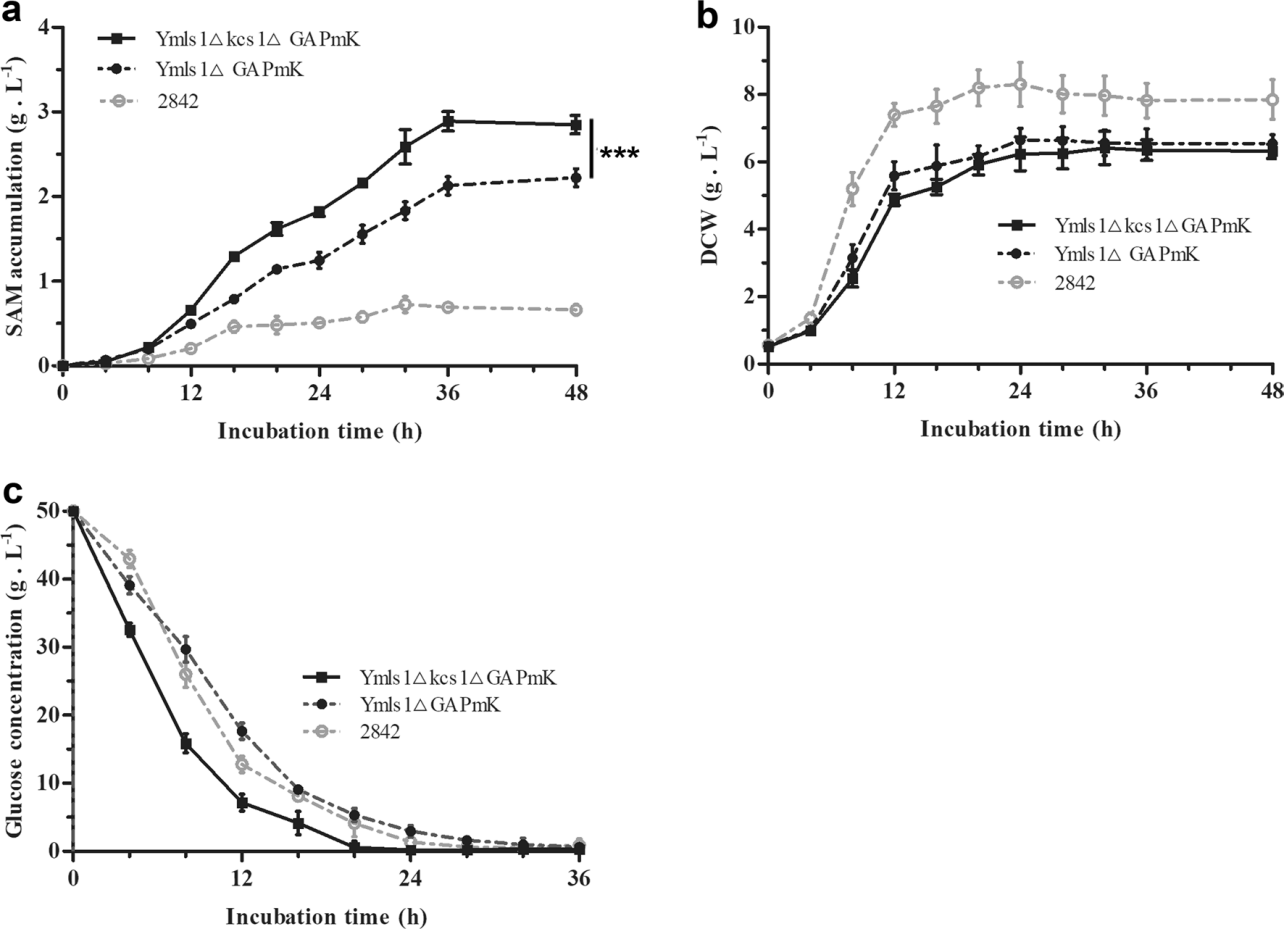
Increased glycolysis by deletion of *kcs1* in the recombinant strain Y*mls1*ΔGAPmK further improved SAM production. **a** The accumulation curve of SAM of the recombinant strain Y*mls1*Δ*kcs1*ΔGAPmK; **b** The growth curve of the recombinant strain Y*mls1*Δ*kcs1*ΔGAPmK; **c** The glucose uptake rate of the recombinant strain Y*mls1*Δ*kcs1*ΔGAPmK. Error bars correspond to standard error of mean (SEM) of the biological replicates (n = 3). *** denotes significant differences between the recombinant strains Y*mls1*Δ*kcs1*ΔGAPmK and Y*mls1*ΔGAPmK, with p < 0.001

For further improving the production of SAM in yeast, *kcs1* was deleted in the high SAM-producing strain Y*mls1*ΔGAPmK reported in our previously study (Chen et al. 2016c), to obtain a new recombinant strain Y*mls1*Δ*kcs1*ΔGAPmK. As shown in Fig. 6, the SAM levels of 2842, Y*mls1*ΔGAPmK and Y*mls1*Δ*kcs1*ΔGAPmK reached 0.66 g L^−1^, 2.22 g L^−1^ and 2.89 g L^−1^. The SAM level of Y*mls1*Δ*kcs1*ΔGAPmK increased 338 % compared to that in 2842 and increased 30.2 % compared to that in Y*mls1*ΔGAPmK (Fig. 6a). The biomass of the mutant strain was lower than those of 2842 and Y*mls1*ΔGAPmK. Conversely, the glucose uptake rate was greater (Fig. 6b and c).

In addition, the ratio of SAM yield to DCW and the ratio of SAM yield to carbon source of the Y*mls1*Δ*kcs1*ΔGAPmK strain reached 455.1 mg g^−1^ and 57.8 mg g^−1^, which represented an increase of 34.1 % and 30.2 %, respectively, compared to those of Y*mls1*ΔGAPmK (Table 4). The results showed that the strategy of increasing glycolysis by the deletion of *kcs1* significantly improved SAM production in recombinant yeast strain Y*mls1*ΔGAPmK.

**Table 4:** Increased glycolysis by deletion *kcs1* in the recombinant strain Ymls1Δkcs1ΔGAPmK further improved SAM production

	2842	Y*mls1*△GAPmK	Y*mls1*Δ*kcs1*ΔGAPmK
SAM (g L^−1^)	0.66 ± 0.06	2.22 ± 0.11***	2.89 ± 0.10***
DCW (g L^−1^)	7.85 ± 0.59	6.54 ± 0.26***	6.35 ± 0.21***
SAM yield to DCW (mg g^−1^)	84.1	339.4***	455.1***
SAM yield to carbon source (mg g^−1^)	13.2	44.4***	57.8***

The symbol *** denotes significant diferences between the recombinant strain and the wild strain, with p < 0.001

### SAM accumulation capacity of Y*kcs1*Δ*mls1*ΔGAPmK by fed‐batch fermentation

To evaluate the SAM accumulation capacity of the Y*mls1*Δ*kcs1*ΔGAPmK strain, fed-batch fermentation was preliminarily scaled up in a 10-L fermenter, according to our previous reports (Chen et al. 2016c). SAM accumulation, reducing sugar concentration, and DCW in the fermentation broth were measured during the entire fermentation process.

**Fig. 7:**
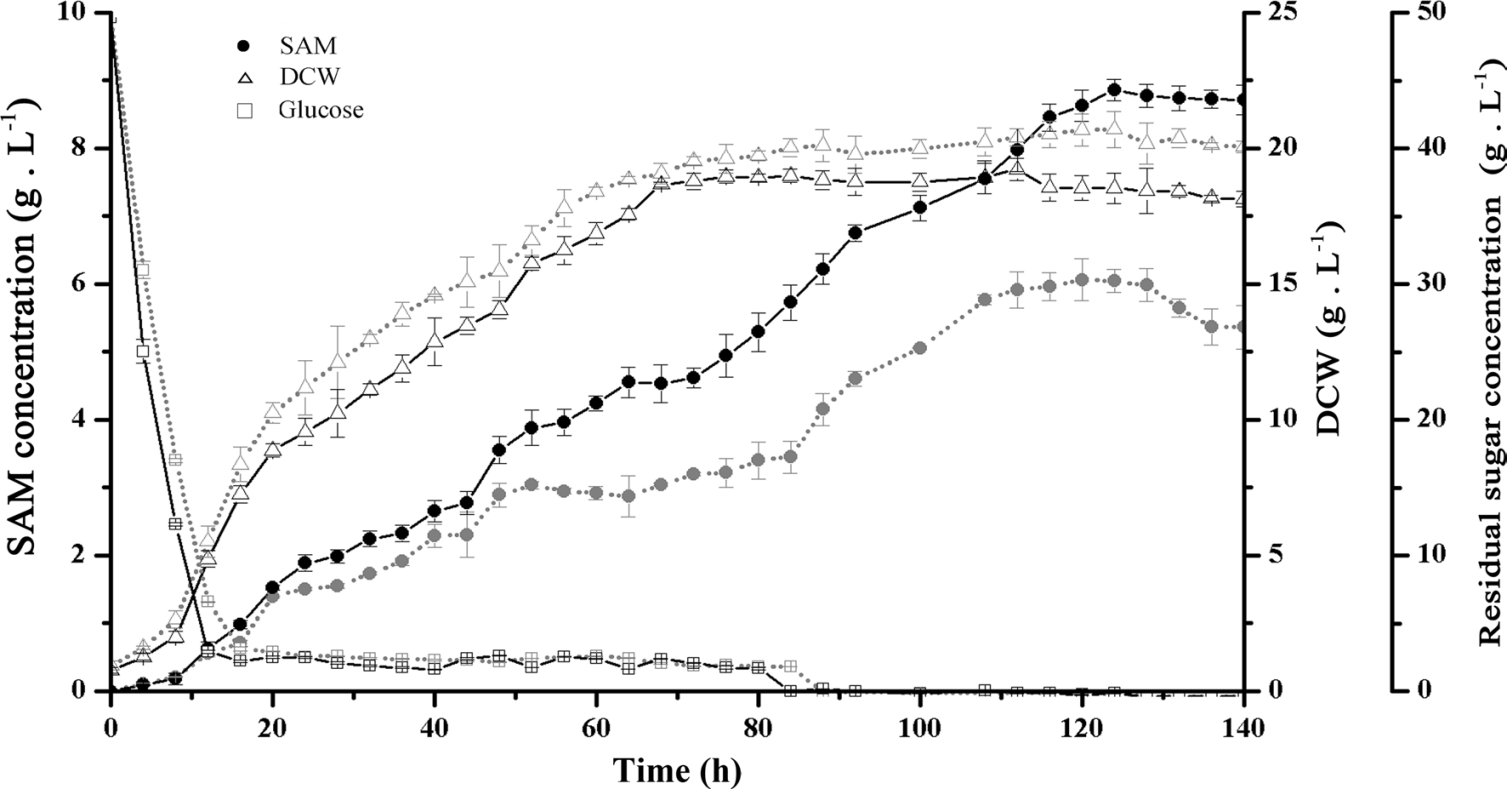
SAM accumulation capacity of Y*kcs1*Δ*mls1*ΔGAPmK by fed-batch fermentation. The black solid line represents Y*mls1*Δ*kcs1*ΔGAPmK strain; The gray dotted line represents Y*mls1*ΔGAPmK strain. Error bars correspond to standard error of mean (SEM) of the biological replicates (n = 3)

Throughout the fermentation process, it was observed that the reducing sugar in the fermentation broth was almost exhausted after 12 h of fermentation. After 12 h of fermentation, to enhance the biomass and maximize the final SAM yield, the molasses was fed into the fermentation broth at a rate of 1.2 g L^−1^ h^−1^ for 72 h (Chen et al. 2016c, 2020). As a result, the growth vitality and efficiency of Y*kcs1*Δ*mls1*ΔGAPmK was maintained, and there was a slight decrease in cell dry weight, with the largest DCW of 19.2 g L^−1^ achieved at 112 h. The accumulation of SAM has been significantly improved, with the highest SAM accumulation of 8.86 g L^−1^ achieved at 128 h which represented an increase of 46.2 % compared to that in Y*mls1*ΔGAPmK (Fig. 7). In addition, the ratio of SAM yield to DCW and the ratio of SAM yield to carbon source of the Y*mls1*Δ*kcs1*ΔGAPmK strain reached 478.4 mg g^−1^ and 13.6 mg g^−1^, which represented an increase of 63.2 % and 46.2 %, respectively, compared to those of Y*mls1*ΔGAPmK (Table 5). The results demonstrated that increasing glycolysis by deletion of *kcs1* is a strategy with great potential for the improvement of the microbial production of SAM.

**Table 5:** The comparations of SAM yields between the recombinant strains Y*mls1*Δ*kcs1*ΔGAPmK and Y*mls1*△GAPmK in a 10-L fermatation tank containing 7 L of O-medium

	Y*mls1*△GAPmK	Y*mls1*Δ*kcs1*ΔGAPmK
SAM (g L^−1^)	6.06 ± 0.31	8.86 ± 0.16***
DCW (g L^−1^)	20.67 ± 0.60	18.52 ± 0.46***
SAM yield to DCW (mg g^−1^)	293.2	478.4***
SAM yield to carbon source (mg g^−1^)	9.3	13.6***

The symbol *** denotes significant diferences between the recombinant strain and the wild strain, with p < 0.001

## Discussion

Glycolysis manages the levels of various interesting metabolites by controlling the supply of glycolytic metabolites. Metabolic reprogramming of glycolysis, which directs the flux of glycolytic metabolites to specific metabolic pathways, would be useful to increase the microbial production of interesting metabolites, such as lipids, amino acids or considerable species of secondary metabolites (Masumoto et al. 2018).

The expressions of several glycolytic genes in yeast are tightly regulated by transcriptional factors GCR1, GCR2 and RAP1 (Saiardi et al. 2004; Santangelo 2006). The promoters of glycolytic genes usually contain a CT-box (GCR1-binding site) and an UAS_RPG_-box (RAP1-binding site). The binding of GCR1 and RAP1 to these sites is facilitated by GCR2 interacting with phosphorylated GCR1. When the intracellular levels of IPs decrease, the absence of pyrophosphorylation of GCR1 activates transcription of the glycolytic genes (Saiardi et al. 2004; Szijgyarto et al. 2011). In short, the expression levels of the glycolytic genes depend on the intracellular levels of IPs. Szijgyarto et al. (2011) deleted *kcs1*, *ipk1* and *arg82*, and investigated gene expressions, enzyme activities and glycolysis metabolism of the mutant. It was demonstrated that the glycolytic flux and the cellular ATP content can be increased by the deletions of genes related to IPs metabolism. Inspired by the study of Szijgyarto et al. (2011), a strategy of increasing glycolysis by altering the metabolism of IPs was carried out for improvement of microbial production of SAM in this study.

Although the metabolism of IPs has diverse roles in phosphate signaling and other important cellular processes, little is known about its function in the biosynthesis of SAM. To explore whether SAM biosynthesis is affected by the metabolism of IPs, the genes related to IPs metabolism *arg82*, *ipk1*, and *kcs1*, were deleted, respectively, in the yeast strain *S. cerevisiae* CGMCC 2842. It was found that the production of SAM was enhanced by the deletion of *arg82* or *kcs1* in yeast but not by the deletion of *ipk1*. The above research confirmed a correlation between SAM biosynthesis and the metabolism of IPs. Analyzing the metabolic pathways of IPs, it was found that the deletion of *arg82* or *kcs1* blocked the anabolism of IPs, but the deletion of *ipk1* did not completely prevent the synthesis of IPs, as PPP-IP_4_, PP-IP_4_ and the (PP)_2_-IP_3_ could still be synthesized (Fig. 1b), which participate in the pyrophosphorylation of GCR1 and repress the transcriptional activities of the glycolytic genes.

The responses of glycolysis to the deletion of *kcs1*, *ipk1* and *arg82* in the *S. cerevisiae* strain were investigated. It was found that compared to the wild type yeast strain 2842, the glucose uptake rates were enhanced, the expression levels of *hxk2*, *pfk1*, *gapdh*, *pgk1* and *pyk* in the *kcs1* deletion strain increased by 77 %, 93 %, 88 %, 186 % and 124 %, and the activities of the related enzymes HK, PFK1, GAPDH, PGK1 and PYK increased by 124 %, 88 %, 65 %, 121 % and 151 %, respectively, in the *kcs1* deletion strain. The same phenomenon was also observed in the *arg82* deletion strain. The results demonstrated that the deletions of *kcs1* and *arg82* increased the expression levels of several glycolytic genes and the relative enzyme activities in yeast. Similar to these above trends, the intracellular levels of ATP, various glycolytic intermediates, and precursor amino acids were significantly enhanced. The results showed that glycolysis was indeed increased by deletions of *kcs1* and *arg82* providing sufficient precursors and energy for the biosynthesis of SAM in yeast and also answered the question of why the glucose uptake rate was enhanced in the mutant strains deleted for *kcs1* and *arg82*. These results of deletions of *kcs1* and *arg82* increased glycolysis and ATP levels in yeast were consistent with the study reported by Szijgyarto et al. (2011). It remains unclear whether there are other factors caused by the deletion of *kcs1* and *arg82* that are also responsible for improvement in SAM production. Further studies are needed.

To intuitively investigate the SAM accumulation capacity of the Y*mls1*Δ*kcs1*ΔGAPmK strain, fed-batch fermentation was preliminarily scaled up in a 10-L fermenter, according to the fermentation conditions and processes of our previous reports (Chen et al. 2016c). It was found that the production of SAM of the Y*mls1*Δ*kcs1*ΔGAPmK strain reached 8.86 g L^−1^, which increased 46.2 % compared to that of the Y*mls1*ΔGAPmK strain. Although it was slightly lower than the current reported maximum production of SAM, this strategy provides a potential theoretical basis for microbial production of SAM or other metabolites that require energy. However, different from the Y*mls1*ΔGAPmK strain, the *kcs1* gene was knocked out for increasing glycolysis in the Y*mls1*Δ*kcs1*ΔGAPmK strain. In addition to energy, the intracellular levels of several glycolytic intermediates and precursor amino acids participating in the metabolism of l-Met were also enhanced, which caused an appropriate balance between the levels of precursor intermediates and ATP for effectively advancing the microbial production of SAM. Thus, if further studies on the optimization of fermentation conditions and the control of the fermentation process are carried out for the new mutant strain Y*mls1*Δ*kcs1*ΔGAPmK, it is believed that a higher level of SAM accumulation will be obtained.

In conclusion, a strategy of increasing glycolysis by altering the metabolism of IPs for improving the production of SAM in yeast is presented. The genes associated with the metabolism of IPs, *arg82*, *ipk1* and *kcs1*, were deleted, respectively, in the yeast strain *S. cerevisiae* CGMCC 2842. It was found that the deletions of *kcs1* and *arg82* increased SAM by 83.3 % and 31.8 %, respectively, compared to that of control. In addition to the improved transcription levels of various glycolytic genes and activities of the relative enzymes, the levels of glycolytic intermediates and ATP were also enhanced. To further confirm the feasibility, *kcs1* was deleted in the high SAM-producing strain Y*mls1*ΔGAPmK to obtain the recombinant strain Y*mls1*Δ*kcs1*ΔGAPmK. The SAM of Y*mls1*Δ*kcs1*ΔGAPmK reached 2.89 g L^− 1^ in a 250-mL flask and 8.86 g L^−1^ in a 10-L fermentation tank, which represented increases of 30.2 % and 46.2 %, respectively, compared to those of Y*mls1*ΔGAPmK. The strategy of increasing glycolysis by deletion *kcs1* and *arg82* improved SAM production in yeast.

## Data Availability

All relevant data are within the manuscript.

## Abbreviations

SAM: S-adenosyl-l-methionine
MAT: Methionine adenosyltransferase
IPs: Inositol pyrophosphates
ATP: Adenosine triphosphate
G3P: 3-Phosphoglycerate
PY: Pyruvate
IP_3_: Inositol trisphosphate
IP_4_: Inositol tetrakisphosphate
IP_5_: Inositol pentakisphosphate
IP_6_: Inositol hexakisphosphate
IP_7_: Diphosphoinositol pentakisphosphate
(PP)_2_-IP_4_: Double-pyrophosphate tetrakisphosphate
IP_8_: Bis-diphosphoinositol tetrakisphosphate
PP-IP_4_: Diphosphoinositol tetrakisphosphate
(PP)_2_-IP_3_: Double-diphosphoinositol trisphosphate
DCW: Dry cell weight
HK: Hexokinase
PFK1: 6-Phosphofructo-1-kinase
GAPDH: Glycerol-3-phosphate dehydrogenase
PGK1: 3-Phosphoglycerate kinase
PYK: Pyruvate kinase
